# Mycoplasma infection aggravates cardiac involvements in Kawasaki diseases: a retrospective study

**DOI:** 10.3389/fimmu.2023.1310134

**Published:** 2024-01-17

**Authors:** Guoyan Lu, Xing Li, Jie Tang, Yuxi Jin, Yang Wang, Kaiyu Zhou, Yifei Li

**Affiliations:** Department of Pediatrics, Ministry of Education Key Laboratory of Women and Children’s Diseases and Birth Defects, West China Second University Hospital, Sichuan University, Chengdu, Sichuan, China

**Keywords:** Kawasaki disease, mycoplasma pneumoniae, prognosis, coronary artery injuries, inflammation

## Abstract

**Background:**

*Mycoplasma pneumoniae* (MP) infection serves as a substantial cofactor in Kawasaki disease (KD) among patients. Although the dominant issue triggering KD has recently focused on MP infection, the complete demonstration of the relationship between MP infection and KD remains elusive. This study endeavors to scrutinize and compare the clinical manifestations and cardiac involvement between MP-triggered KD and non-infection–associated KD.

**Method:**

This retrospective study (2023-039, approved by the Institutional Review Board of West China Second University Hospital of Sichuan University) encompassed 247 consecutive patients diagnosed with KD between June 2017 and December 2022. Patients were categorized into two groups: the MP group (n = 38) and the non-MP group (n = 209). Univariable analysis was utilized to discern differences in clinical features, severity of inflammation, and initial or persistent cardiac complications between the two groups.

**Results:**

The MP group exhibited a more intricate clinical profile compared with the non-MP group, characterized by prolonged hospital stays, a higher incidence of incomplete KD, and elevated comorbidities. In addition, MP infection correlated with severe hematological disorders, coagulation dysfunction, and myocardial injuries. Our findings revealed that MP infection led to prolonged inflammation after initial treatment with intravenous immunoglobulin. Although initial cardiac assessments failed to discern disparities between the two groups, MP infection notably exacerbated coronary artery aneurysms (CAAs), resulting in sustained dilation.

**Conclusions:**

Recognizing MP infection as a significant infectious factor associated with KD is imperative. In patients with KD, MP infection significantly prolongs inflammation and causes hematological disturbances during the initial treatment phase. Moreover, the presence of MP infection exacerbates the progression of CAAs and myocardial injuries during the subacute phase of KD, consequently contributing to the persistence of CAAs.

## Introduction

Kawasaki disease (KD) is defined as acute, febrile, and systemic vasculitis in children, which primarily affects medium-sized blood vessels with a tropism for the coronary arteries, resulting in endothelium dysfunction (1). KD has been considered as an acute systemic vasculitis that predominantly affects patients <5 years of age. In the absence of an available and affordable diagnostic test, detailed clinical history and physical examination remain fundamental to the diagnosis of KD. Although the etiological factors of KD remain unknown, infections have been suggested as one of the dominant causes to trigger KD (2, 3). Notably, a study by Kang et al. reported the nonpharmaceutical interventions reduced the prevalence of KD and COVID-19, indicating that infections with viruses or microorganisms contributed to the onset of KD (4). In addition, a study conducted by Rand et al. concluded that the lockdown and social distancing during the COVID-19 pandemic led to a marked decrease in respiratory virus circulation, providing an “experiment of nature” to determine whether KD would decline in parallel (2). However, they found that it was not paralleled by a comparable decrease in the incidence of KD. Furthermore, a national survey from United States suggested that social behavior is associated with exposure to the agents that trigger KD, which is consistent with a respiratory portal of entry for the agents (5). Therefore, it was of great significance to determine the association between KD and common infectious agents, and whether combined infection would result in prolonged injuries to myocardium or coronary arteries.

*Mycoplasma pneumoniae* (MP) is considered the main pathogen of pneumonia in school-age children. An increasing number of cases of MP pneumonia have been reported in children <5 years old (6). Mycoplasma infections can manifest in a variety of clinical presentations, ranging from respiratory tract infections to extrapulmonary manifestations. The symptoms of extrapulmonary mycoplasma infection in children are non-specific and are often similar or identical to those of KD (7). Therefore, MP is suggested to be one of the infectious sources that triggers the KD immune response (8). Hu et al. compared the MP infection–triggered KD with KD not associated with MP (7). They found that the complement C3 and CD4/CD8 ratio were significantly increased in MP-triggered KD compared with that in KD alone and that both C3 and polyclonal CD4(+) T lymphocytes may be activated in the patients with MP-triggered KD (7).

MP infection is associated with a multisystem inflammatory syndrome (MIS) (9). As it was initially reported in children as MIS due to COVID-19 with Kawasaki-like disease, the association between MP infection and KD onset has been recently recognized (10). In addition, the rashes and mucositis induced by MP infection have been considered as a KD-like presentation (11). Huang et al. reviewed the most recent studies and concluded that patients with KD with MP infection presented with a high proportion of cardiac involvement, which could also be associate with attacks of cholestatic hepatitis (12). Furthermore, they found the early treatment with intravenous immunoglobulin (IVIG) would be beneficial for such patients. In addition, MP infection had been reported to increase the risk of recurrent KD in a Chinese cohort (13). This immune mechanism was very similar to KD (14).

Therefore, it is important to evaluate the impact of MP infection on the clinical presentation, characteristics, and prognosis of KD, especially regarding cardiac involvement. Herein, we aimed to demonstrate the differences between MP-triggered KD and non-infection associated KD. This study conducted a retrospective analysis of the clinical data from 247 pediatric patients who were diagnosed with KD, of which 38 patients were found to have MP infection. The clinical characteristics and short-term clinical outcomes between patients with and without MP infection have been assessed.

## Methods

### Patient population

All patients were enrolled for in this retrospective study from June 2017 to December 2022 at Sichuan University West China Second Hospital. This study was approved by Ethical Committee and Institutional Review Board of the West China Second Hospital of Sichuan University (2023–039). Written informed consents were obtained from the participants’ parents to have their medical records included in medical research. No information could be used to identify individual participants.

### Inclusion and exclusion criteria

The inclusion of patients with complete KD was based on the criteria defined by the 2017 American Heart Association (AHA) (15): guidelines as fever lasting for at least 5 days, plus four of the following five clinical criteria: 1) extremity changes; 2) rash; 3) conjunctivitis; 4) oral changes; and 5) cervical lymphadenopathy (>1.5 cm in diameter, usually unilateral). Incomplete KD was also diagnosed based on the criteria defined by the 2017 AHA guidelines.

MP infection was diagnosed on the basis of the criteria defined as follows: respiratory samples (pharyngeal swabs, sputum, and bronchoalveolar lavage fluid) that underwent polymerase chain reaction (PCR), leading to the detection of a positive results for MP RNA, or MP-IgM antibodies detected by enzyme-linked immunosorbent assay (ELISA) ≥1.0, or the MP-IgM or MP-IgG antibody titers increased or decreased by four-fold during the acute and recovery stages.

The exclusion criteria were as follows: 1) the patient’s blood culture was positive; 2) the patient had been diagnosed with a connective tissue disease, including systemic lupus erythematosus and juvenile idiopathic arthritis; 3) certain fevers were excluded, such as rheumatic fever and fever with unknown origin; 4) patients who had not been treated according to the clinical guidelines; 5) prior use of anticoagulants or antiplatelet medications before the onset of KD; 6) history of cardiac surgery; 7) suspicion of myocarditis prior to KD onset (6); use of glucocorticoids prior to IVIG administration; 8) use of monoclonal antibodies, including tumor necrosis factor–α and interleukin-6 antibodies; 9) diagnosis of macrophage activation syndrome or hemophagocytic lymph histiocytosis secondary to KD; 10) new onset of coronary artery lesions following persistent coronary artery aneurysms (CAAs); 11) the absence of echocardiographic records for ≥ 1 months; 12) influenza A or B infection detected by PCR; 13) COVID-19 infection; 14) HIV infection; and 15) certain bacterial infections that had been addressed.

### Therapeutics and follow-up procedure

The initial treatment received by all patients followed the strict guideline, which involved IVIG (2 g/kg given as a single intravenous infusion) combined with 30–50 (mg/kg)/day high-dose aspirin. After maintaining normal body temperature for 3 days, the aspirin dose was reduced to 3–5 (mg/kg)/day and continued for 6–8 weeks. For patients who did not respond to the second IVIG treatment, a supplementary therapy with methylprednisolone at a dose of 20–30 mg/kg was administered intravenously for 3 days, followed by oral prednisone at a dose of 1–2 mg/kg for 2–3 weeks. If CAAs were present, then aspirin therapy continued until the coronary artery recovered to normal. In cases where CAAs reached a moderate size, clopidogrel (1 mg/kg) was administered orally in addition to aspirin. For patients with concurrent MP infection, those with mild symptoms received oral azithromycin at a dose of 10 (mg/kg)/day for 3 days, whereas severe cases continued treatment for 5–7 days.

All patients were divided into the MP infection group (MP group) and non-MP infection group (non-MP group). The clinical profiles of the two groups were compared. Laboratory data were collected before and after IVIG initiation and completion of treatment. Echocardiography was performed on days 5–10 of fever in all patients before initial treatment. After discharge, all patients were followed up at the outpatient clinic for a minimum of 2 months and underwent routine cardiac ultrasound examinations.

The commencement of fever was designated as the day of onset (day 1) of KD. Resistance to IVIG was characterized by the persistence or recurrence of fever (oral temperature ≥38.0°C) or the presence of other clinical manifestations of KD for a duration of at least 36 h but no longer than 7 days after the initial IVIG treatment. Hospital discharge occurred once the patient had maintained a normal body temperature for >48 h, and their hematological examination results had returned to normal. Subsequently, follow-up commenced from the day of hospital discharge. All study participants underwent echocardiographic assessments at 2 weeks, 1 month, 1.5 months, and 2 months; the data were collected from the conclusion of the subacute phase.

### Clinical outcomes and echocardiographic evaluation

All clinical parameters were documented in detail along with 2 months of follow-up. First, the basic clinical characteristics and hematological examination results of the participants were recorded. The initial hematological examinations following the first IVIG administration were also noted. Furthermore, liver function, kidney function, coagulation, and myocardial injury biomarkers were also recorded. Thereafter, univariable analysis was performed between the MP and non-MP groups to illustrate the differences among the initial clinical parameters, therapeutic efficacies, CAAs, and recovery of the coronary artery injuries.

Two extensively trained pediatric physicians conducted all echocardiographic assessments. These evaluating physicians were unaware of the clinical presentations of the participants. The initial echocardiographic evaluation transpired before the administration of IVIG, whereas the subsequent evaluation occurred either during the subacute phase or prior to hospital discharge. We defined coronary artery morphology and luminal dilation, as represented by the aneurysm size, based on the most severe condition observed between the acute and subacute phases of KD. A minimum of two echocardiographic assessments were executed to meet the essential criteria for the 1-month follow-up. Furthermore, the first instance of a pre-existing CAA regression to a normal size was recorded as the regression time. If a CAA persisted for a duration of 12 months, then it was categorized as a persistent lesion. Following the guidelines set forth by the Japanese Circulation Society (16), CAAs were characterized by a coronary artery branch internal luminal diameter >3 mm in children <5 years of age, an arterial diameter >4 mm in children aged 5 years and older, or when an arterial segment measured 1.5 times the size of its adjacent segment. These CAAs were further classified as small aneurysms (exhibiting localized dilatation with an internal diameter of 4 mm or less), medium aneurysms (aneurysms with an internal diameter ranging from over 4 mm to less than 8 mm), and giant aneurysms (aneurysms with an internal diameter of 8 mm or more).

### Statistical analysis

The SPSS 22.0 (SPSS Inc. Chicago, Illineois, United States of America) software package was used for data analysis. Quantitative data were presented as the mean ± standard deviation and median with range, whereas qualitative data were expressed as n. Differences between the two groups were assessed using the independent t-test or Mann–Whitney U-test for continuous variables and the chi-squared test or Fisher’s exact test for categorical variables. p-values <0.05 were defined as statistically significant.

## Results

### MP infection induces severe hematological disorders and myocardial injury

This study included 247 patients diagnosed with KD, including 38 cases (15.4%) combined with MP infection and 209 cases (84.6%) without MP infection. Of the MP group, 18 (18/38, 47%) patients tested positive with PCR, 26 (26/38, 68%) patients were serological antibody positive, and eight patients had confirmed MP infection by both approaches. Clinical hematological manifestations and laboratory data of both groups were presented in **Supplementary Table 1.**

In general, there were no differences in sex (p = 0.593), body weight (p = 0.336), and age (p = 0.098) between the two groups. The MP group exhibited a more complicated clinical status compared with the non-MP group. MP infection resulted in a longer hospital stay (7.97 ± 3.98 days vs. 5.49 ± 3.50 days; p = 0.000), a higher incidence of incomplete KD (26.32% vs. 9.57%; p = 0.012), and increased ratio of comorbidities in the respiratory system (65.79% vs. 38.28%; p = 0.002) and neurological system (28.95% vs. 13.40%; p = 0.027), whereas there was no significant difference in the ratio of complications of digestive, muscular, and urinary systems (**Table 1**).

**Table 1:** Clinical characteristics of patients with KD with or without MP infection.

Variables	MP (n = 38)	non-MP (n = 209)	Sig.
Gender			
Female	18 (47.37%)	87 (41.63%)	0.593
Male	20 (52.63%)	122 (58.37%)	
Age (years)	3.94 ± 2.46	3.27 ± 2.24	0.098
Weight (kg)	16.08 ± 6.51	14.89 ± 7.04	0.336
Hospital stays (days)	7.97 ± 3.98	5.49 ± 3.50	0.000
Fever duration (days)	5.47 ± 3.36	6.33 ± 1.73	0.446
Incomplete KD	10 (26.32%)	20 (9.57%)	0.012^*^
Respiratory complications	25 (65.79%)	80 (38.28%)	0.002^*^
Neurological complications	11 (28.95%)	28 (13.40%)	0.027^*^
Digestive system complications	15 (39.47%)	81 (38.76%)	1.000
Muscle system complications	7 (18.42%)	28 (13.40%)	0.448
Urinary system complications	0 (0.00%)	2 (0.96%)	1.000

*p < 0.05.

**Figure 1:**
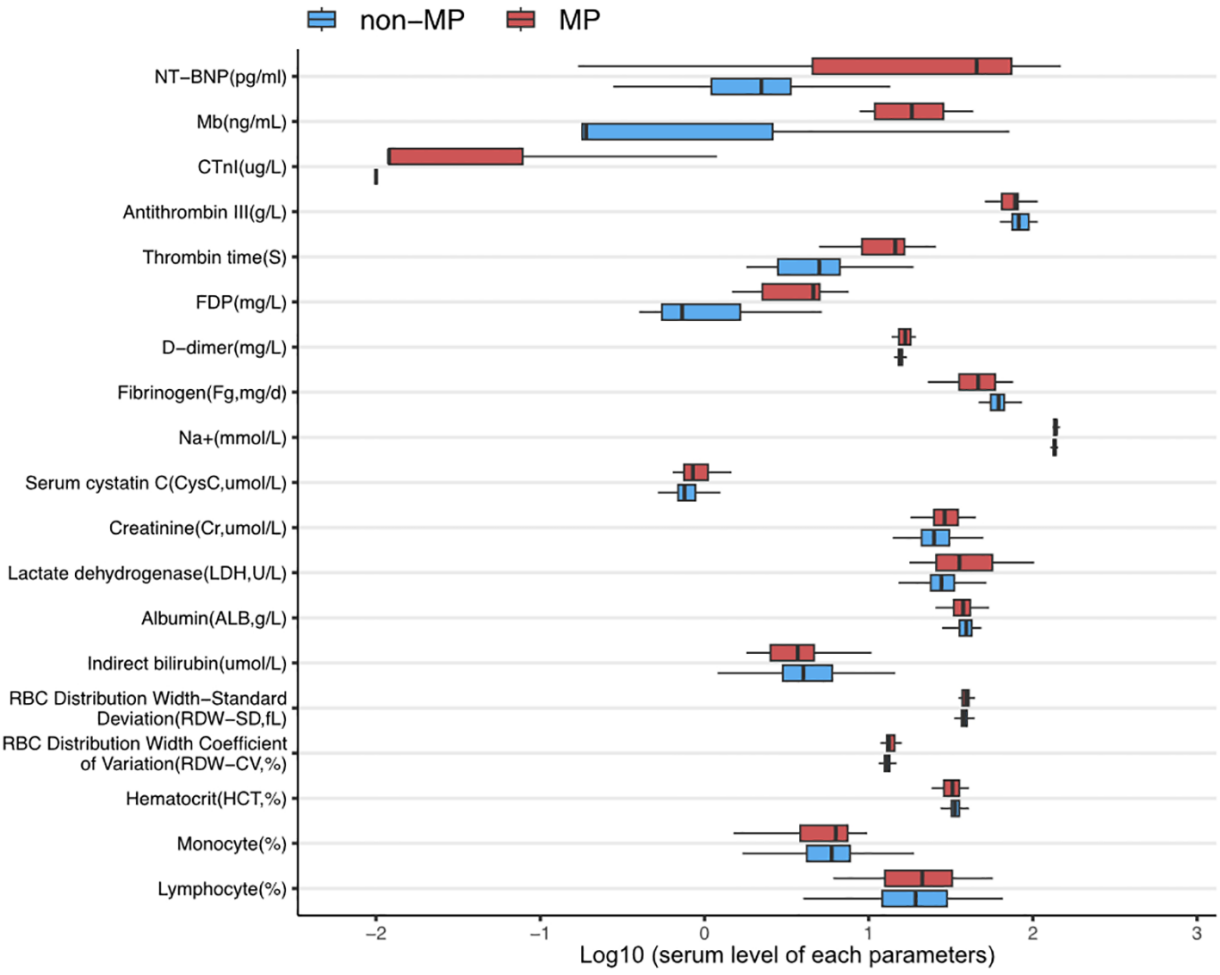
The expression levels of parameters with significant differences between MP infection and non-infection groups. All data were presented in log10(value).

In addition, as shown in **Figure 1**, there were several parameters that differed between groups in the hematological examinations, such as an elevated ratio of lymphocytes (30.96 ± 46.77 vs. 21.92 ± 12.96; p = 0.024), ratio of monocytes (24.65 ± 17.53 vs. 6.64 ± 5.16; p = 0.036), red cell distribution width–standard deviation (RDW-SD, fL; 39.28 ± 3.20 vs. 37.96 ± 2.74; p = 0.001), red cell distribution width–coefficient of variation (RDW-CV, %; 13.75 ± 1.52 vs. 13.09 ± 1.05; p = 0.001), and decreased hematocrit (HCT, %; 33.23 ± 4.59 vs. 33.70 ± 3.05; p = 0.015). In addition, MP infection induced a reduction in indirect bilirubin (µmol/L; 3.69 ± 2.84 vs. 4.87 ± 2.90; p = 0.033) and albumin (g/L; 37.01 ± 6.74 vs. 38.96 ± 4.78; p = 0.035), in addition to an increase in lactate dehydrogenase (U/L; 434.11 ± 220.69 vs. 307.94 ± 142.43; p = 0.000), creatinine (µmol/L; 30.70 ± 8.73 vs. 26.81 ± 7.63; p = 0.006), and serum cystatin C (µmol/L; 0.93 ± 0.26 vs. 0.80 ± 0.18; p = 0.012). Furthermore, disorders in coagulation function were identified in the MP group, including a significant decrease in fibrinogen (mg/d; 462.83 ± 175.95 vs. 613.71 ± 119.60; p = 0.000) and elevated D-dimer (mg/L; 6.45 ± 8.36 vs. 1.21 ± 1.10; p = 0.009), fibrin degradation products (mg/L; 19.74 ± 23.22 vs. 9.95 ± 14.25; p = 0.172), thrombin time (S; 16.64 ± 1.68 vs. 15.61 ± 0.78; p = 0.000), and antithrombin III (g/L; 65.31 ± 30.71 vs. 83.78 ± 14.43; p = 0.034).

Most importantly, MP infection leads to more severe myocardial injuries, with the MP group showing significantly elevated biomarkers, including cardiac troponin (CTnI, µ g/L; 0.15 ± 0.31 vs. 0.021 ± 0.028; p = 0.025), myoglobin (Mb, ng/mL; 20.43 ± 11.86 vs. 7.20 ± 17.67; p = 0.018), and N-terminal pro B-type natriuretic peptide (NT-proBNP, pg/mL; 5152.92 ± 5536.38 vs. 925.95 ± 2368.25; p = 0.019).

However, regarding other parameters, including white blood cell (WBC) count, neutrophil percentage, absolute neutrophil count, red blood cell count, hemoglobin, platelet count, and C-reaction protein (CRP) levels, no significant differences were observed between the two groups. When comparing the treatment outcomes between the two groups, there was no significant difference in the IVIG resistance (p = 0.260) and the additional administration of steroid pulse therapy (p = 0.399).

### Prolonged hematological disorders are caused by MP infection

An initial hematological examination was performed after the first dose of IVIG, and the results are shown in **Table 2**. Notably, the MP group demonstrated an increased WBC (%; 10.77 ± 5.48 vs. 8.93± 4.15; p = 0.021), neutrophil percentages (%; 52.86 ± 24.03 vs. 44.63 ± 18.72; p = 0.021), and absolute neutrophil counts (×10^9^/L; 6.52 ± 5.08 vs. 4.29 ± 3.45; p = 0.001) compared with the non-MP group. In addition, the hemoglobin and HCT levels were significantly lower in the MP group than that in the non-MP group (p < 0.05) after the initial IVIG therapy, suggesting prolonged hematological disturbances. However, there was no significant difference in the CRP level between the two groups after the initial IVIG administration (p = 0.828).

**Table 2:** Laboratory parameters after the first intravenous immunoglobulin therapy of patients with KD with or without MP infection.

Variables	MP (n = 38)	non-MP (n = 209)	Sig.
White blood cell (×10^9^/L)	10.77 ± 5.48	8.93 ± 4.15	0.021^*^
Neutrophils (%)	52.86 ± 24.03	44.63 ± 18.72	0.021^*^
Lymphocyte (%)	44.80 ± 23.12	41.44 ± 16.51	0.464
Monocyte (%)	4.45 ± 3.43	8.25 ± 2.81	0.251
Absolute neutrophil count (×10^9^/L)	6.52 ± 5.08	4.29 ± 3.45	0.001^*^
Red blood cell (×10^12^/L)	3.91 ± 0.48	4.04 ± 0.44	0.131
Hemoglobin (g/L)	103.00 ± 12.25	107.28 ± 10.75	0.032^*^
Hematocrit (HCT, %)	30.86 ± 3.60	32.07 ± 3.12	0.037^*^
RBC distribution width–coefficient of variation (RDW-CV, %)	14.21 ± 1.86	13.05 ± 1.01	0.000^*^
Red cell distribution width–standard deviation (RDW-SD, fL)	40.08 ± 3.28	37.90 ± 4.99	0.013^*^
Platelet (×10^9^/L)	433.56 ± 161.91	446.89 ± 137.90	0.603
CRP (mg/L)	46.16 ± 52.61	37.59 ± 34.73	0.828
IVIG resistance (%)	9(23.68%)	71(33.97%)	0.260
Steroid additional therapy (%)	6(15.79%)	50(23.92%)	0.399

*p < 0.05; CRP, C-reactive protein; KD, Kawasaki diseases; IVIG, intravenous immunoglobulin; MP, Mycoplasma pneumoniae; RBC, red blood cell.

### MP infection leads to persistent cardiac involvement

The initial cardiac evaluation results are documented in **Table 3**. The cardiac ultrasound was completed before the first administration of IVIG and aspirin. Interestingly, cardiac involvement did not present a significant difference between groups regarding cardiac enlargement, pericardial effusion, valvar regurgitation, and coronary artery dilation at the initial cardiac evaluation. However, the average diameters of left main coronary artery (mm; 3.14 ± 1.14 vs. 2.50 ± 0.62; p = 0.006) and left anterior descending artery (mm; 2.60 ± 1.01 vs. 1.99 ± 0.46; p = 0.001) in the MP group were significantly larger those in the non-MP group, indicating that MP infection did not aggravate the initial cardiac involvements before treatment.

**Table 3:** Initial cardiac ultrasound results of patients with KD with or without MP infection.

Variables	MP (n = 38)	Non-MP (n = 209)	Sig.
Abnormal initial cardiac ultrasound	13 (34.21%)	78 (37.32%)	0.855
Cardiac enlargement	6 (15.79%)	14 (6.70%)	0.097
Pericardial effusion	1 (2.63%)	10 (4.78%)	1.000
Valvar regurgitation	8 (21.05%)	23 (11.00%)	0.108
Coronary artery lesions	7 (18.42%)	37 (17.70%)	1.000
Left coronary artery (mm)	3.14 ± 1.14	2.50 ± 0.62	0.006^*^
Left anterior descending artery (mm)	2.60 ± 1.01	1.99 ± 0.46	0.001^*^
Left circumflex artery (mm)	1.83 ± 0.59	1.58 ± 0.62	0.299
Right coronary artery (mm)	2.88 ± 2.01	2.40 ± 2.30	0.549

*p < 0.05; KD, Kawasaki diseases; MP, Mycoplasma pneumoniae.

However, in the short-term follow-up at the end of the subacute phase of KD duration, the cardiac evaluation demonstrated higher incidence and more severe conditions regarding cardiac involvement (**Table 4**). Although there was no significant difference in the ratio of general cardiac abnormalities between two groups, MP infection resulted in significantly more cases with cardiac enlargement (13.16% vs. 2.87%; p = 0.015). Most importantly, the coronary artery lesions remained in a large proportion of the MP group (21.05% vs. 7.18%; p = 0.012). In addition, the average diameters of the left main coronary artery (mm; 3.33 ± 0.93 vs. 2.63 ± 0.86; p = 0.010) and left circumflex artery (mm; 1.97 ± 0.53 vs. 1.56 ± 0.50; p = 0.018) in the MP group were significantly larger than those in the non-MP group. Furthermore, the average diameter of the right coronary artery in the MP group increased compared with that in the non-MP group (mm; 3.28 ± 1.77 vs. 2.34 ± 0.85; p = 0.003). Right coronary artery injuries were found to increase in MP infection patients during the 2-month follow-up. Therefore, these results revealed the MP infection aggravated the coronary artery injuries and converted into persistent cardiac involvement.

**Table 4:** Cardiac ultrasound after 2 months of follow-up in patients with KD with or without MP infection.

Variables	MP (n = 38)	Non-MP (n = 209)	Sig.
Abnormal initial cardiac ultrasound	13 (34.21%)	56 (26.79%)	0.432
Cardiac enlargement	5 (13.16%)	6 (2.87%)	0.015^*^
Pericardial effusion	2 (5.26%)	10 (4.78%)	0.113
Valvar regurgitation	3 (7.89%)	24 (11.48%)	0.777
Coronary artery lesions	8 (21.05%)	15 (7.18%)	0.012^*^
Left coronary artery (mm)	3.33 ± 0.93	2.63 ± 0.86	0.010^*^
Left anterior descending artery (mm)	2.74 ± 1.06	2.15 ± 1.07	0.106
Left circumflex artery (mm)	1.97 ± 0.53	1.56 ± 0.50	0.018^*^
Right coronary artery (mm)	3.28 ± 1.77	2.34 ± 0.85	0.003^*^

*p < 0.05; KD, Kawasaki diseases; MP, Mycoplasma pneumoniae.

## Discussion

To date, several studies have substantiated the co-occurrence of MP infection and KD. In an investigation by Lee et al., high titers of anti-MP antibody were detected in 22.2% of 54 patients with KD (17). The incidence of KD appeared to surge in 2020, coinciding with a notable increase in MP infections (18). Notably, KD often presents with nonspecific symptoms, including vomiting, diarrhea, abdominal pain, and cough, before being diagnosed (19). Numerous infectious agents, encompassing bacteria, viruses, and atypical pathogens, such as MP, have sporadically been isolated from patients with KD (20–22). Our study identified MP infections in 15.4% of patients with KD, underscoring the potential concurrent occurrence of MP infections in children with KD.

Regarding age distribution, our findings indicated no statistically significant difference between the two groups (p = 0.098), which contrasts with prior investigations. Previous research has consistently reported that the MP-infected group tended to be older than the non-MP group (6, 7, 17). This discrepancy might be attributed to factors such as the isolation measures enforced during the COVID-19 epidemic, prolonged close contact between young children and their parents, and the age-related susceptibility of infants to MP infections. Ding et al. also supported this perspective in their research and postulated that younger infants infected with MP might be more predisposed to developing KD (18). Obviously, higher MP infection was associated with higher ration of pulmonary infection in the studied cohort. Although the exact underlying mechanism remains elusive, Kano et al. proposed that dysregulation of the innate immune response and immaturity of the host’s adaptive immunity might contribute to this phenomenon (23).

In the study, although there was no statistically significant difference in the duration of fever between the two groups, which is consistent with findings from previous studies (6, 8, 17), we did observe notable distinctions in blood parameters in the MP group, even after IVIG treatment. Specifically, the MP group exhibited higher WBC counts, absolute neutrophil counts, lower hemoglobin levels, and higher RDW values when compared with the non-MP group. These variances suggest that MP infection may exert distinct effects on these parameters, indicating that KD combined with MP infection is associated with a more pronounced inflammatory response and increased red blood cell involvement.

MP infections can give rise to extrapulmonary manifestations encompassing various organ systems, including the skin, brain, kidneys, musculoskeletal system, digestive system, and even the hematological system (24). Our study revealed that the MP group displayed more pronounced multiple organ damage, manifesting as respiratory symptoms (65.79% vs. 38.28%, p = 0.002), neurological complications (28.95% vs. 13.40%; p = 0.027), myocardial injury, and abnormal coagulation function. Although the liver and kidney function in the MP group remained within the normal limits, their measured values were significantly higher than those of the non-MP group, suggesting that MP infection can induce substantial extrapulmonary damage (14, 24, 25). Moreover, our results demonstrated that the MP infection induced higher incidence of neurological system complications, which indicated that the inflammation responses for MP would potentially contribute to neurological injuries (26, 27). The pathogenesis of MP-induced extrapulmonary manifestations typically involves three possible mechanisms: direct damage resulting from invasion or locally produced inflammatory cytokines, indirect damage mediated by the immune system, and vascular occlusion due to vasculitis or thrombosis (28, 29). MP infection could potentially act as a triggering factor for autoimmune reactions and the development of multiple organ damage and KD vasculitis. Investigating MP infection in the context of KD etiology may yield valuable insights into the pathogenesis of KD. The factors elucidating the prolonged hospital stay (7.97 ± 3.98 days vs. 5.49 ± 3.50 days; p = 0.000) and the increased incidence of incomplete KD (26.32% vs. 9.57%; p = 0.012) in the MP group in comparison with that in the non-MP group may be attributed to several causative elements. MP infections have the potential to induce multifaceted systemic dysfunction, potentially contributing to more intricate clinical manifestations in patients with KD. Given the diverse clinical presentations identified among KD cases associated with MP infection, these manifestations differ from typical KD symptoms, possibly accounting for the heightened occurrence of incomplete KD. However, there were no significant differences in IVIG resistance (p = 0.260) and the use of steroid pulse therapy (p = 0.399).

Regarding the results of the cardiac evaluation, we found the following (1): During the initial assessment, most cardiac parameters did not exhibit significant differences between the two groups, except for specific coronary artery sizes. These findings align with previous research outcomes (30–33) (2). A noteworthy proportion of patients in the MP group (21.05%) experienced CAAs after subacute phase, which is a distinctive feature of this subgroup (3). Over the 2-month follow-up period, notable differences in the cardiac status emerged between the two groups. Cardiac enlargement was more prevalent in the MP group (13.16% vs. 2.87%, p = 0.015), and CAAs were also more frequently observed in this group (21.05% vs. 7.18%; p = 0.012). Conversely, the non-MP group displayed a significant decrease in individuals with cardiac enlargement compared with the initial assessment, whereas the MP group exhibited little change in this regard. Furthermore, measurements of both the right coronary artery and left coronary artery increased in the MP group. These findings suggest that MP infection may act as a potential cofactor in the development of CAAs in KD, causing persistent CAAs, possibly through superantigen-mediated vascular changes (34). It is important to note, however, that three other studies (6, 17, 35) did not identify significant differences in coronary arterial lesions between the two groups. Therefore, larger study samples are needed to conclusively verify the relationship between MP infection and CAAs.

While inflammation of the coronary arteries is a central aspect of KD, the condition is characterized by systemic inflammation affecting medium-sized arteries throughout the body and multiple organs and tissues during the acute febrile phase. Myocarditis is often observed in this context, and pericarditis and myocarditis typically result from subacute chronic inflammation, often concentrated around the coronary arteries (15). In our study, parameters of myocardial injury, including CTnI, Mb, and NT-proBNP, were significantly higher in the MP group compared with that in the non-MP group. This suggests that the MP group experienced more pronounced acute and chronic myocarditis, as well as more persistent vascular inflammation and coronary artery damage, even when there were no significant differences observed between the two groups in terms of IVIG resistance. According to the Kobayashi scoring system and other retrospective studies, high-risk factors for IVIG resistance in KD include hyponatremia, hypoalbuminemia, elevated aspartate transaminase, age, and sex. IVIG resistance has been considered as a critical issue in KD management, which is associated with the repeat administration of IVIG and additional steroid therapy. However, some studies demonstrated that IVIG resistance only determines the comorbidities beyond cardiac involvement. In addition, in our previous study on KD, we failed to draw a certain association between coronary dilations or myocarditis with IVIG resistance. In this study, there was no significant difference in the CRP level between the MP and non-MP groups, which decided the possibility of IVIG resistance. Thus, we considered that the expression of cytokines could be inhibited after the initial IVIG treatment, whereas the activation of immune cells would persist in the MP group. Indeed, activated immune cells have been proven to be involved in myocardial injury. The above mechanisms could explain why MP infection induced more prolonged inflammation and the increased possibility of cardiac involvement without an increase in IVIG resistance. Such explanation only provides a potential association between more severe myocardial injuries and prolonged inflammation activities without measurement of the cytokines.

Several limitations are associated with this retrospective study. Firstly, this is a single-center retrospective study, which limits its scope and the generalizability of the findings. Secondly, not all children with KD underwent pathogen testing. Thirdly, serological detection of MP antibodies has its limitations. Finally, CAAs in KD typically begin to develop 7–10 days after illness onset but can progress at any stage. Thus, a long-term follow-up of CAAs is essential for a comprehensive assessment of their development and progression.

## Conclusion

MP infection should be recognized as a significant infectious factor associated with KD. Notably, MP infection contributes to the prolonged presence of hematological disturbances in patients with KD during the initial stages of treatment. Although there were no disparities in the incidence of cardiac involvement observed prior to the initiation of treatment, the presence of MP infection exacerbates the development of CAAs and myocardial injury in the subacute phase of KD through increased activation of the inflammatory responses. Thus, the management of MP infection should be emphasized in KD treatment, especially in low-income regions.

## Data availability statement

The original contributions presented in the study are included in the article/**Supplementary Material**, further inquiries can be directed to the corresponding author/s.

## Ethics statement

The studies involving humans were approved by Ethical Committee of the West China Second Hospital of Sichuan University. The studies were conducted in accordance with the local legislation and institutional requirements. Written informed consent for participation in this study was provided by the participants' legal guardians/next of kin. 

## Author contributions

GL: Conceptualization, Data curation, Investigation, Methodology, Validation, Writing – original draft. XL: Data curation, Formal Analysis, Investigation, Methodology, Software, Writing – original draft. JT: Data curation, Investigation, Writing – original draft. YJ: Investigation, Methodology, Writing – original draft. YW: Data curation, Formal Analysis, Investigation, Methodology, Project administration, Supervision, Writing – review & editing. KZ: Methodology, Project administration, Software, Supervision, Validation, Visualization, Writing – review & editing. YL: Conceptualization, Formal Analysis, Funding acquisition, Investigation, Methodology, Project administration, Resources, Software, Supervision, Visualization, Writing – original draft, Writing – review & editing.
