# Supplementary material for: Mycoplasma infection aggravates cardiac involvements in Kawasaki diseases: a retrospective study
Source: Front Immunol. 2024 Jan 17;14:1310134. doi: 10.3389/fimmu.2023.1310134 (PMC10832023; doi:10.3389/fimmu.2023.1310134)
Supplement: Supplementary file 1 [file Table_1.docx]

**Supplementary Table 1.** Clinical hematological characteristics of KD patients with or without MP infection

| Variables | MP (n = 38) | non-MP (n=209) | Sig. |
| --- | --- | --- | --- |
| White blood cell (×10^9^ /L) | 14.46±6.50 | 14.07±4.82 | 0.665 |
| Neutrophils (%) | 68.16±15.05 | 69.05±16.67 | 0.761 |
| Lymphocyte (%) | 30.96±46.77 | 21.92±12.96 | 0.024^*^ |
| Monocyte (%) | 24.65±17.53 | 6.64±5.16 | 0.036^*^ |
| Absolute neutrophil count (×10^9^ /L) | 10.22±5.89 | 9.78±4.40 | 0.602 |
| Red blood cell (×10^12^ /L) | 4.08±0.53 | 4.88±9.06 | 0.588 |
| Hematocrit (HCT, %) | 32.23±4.59 | 33.70±3.05 | 0.015^*^ |
| RBC Distribution Width Coefficient of Variation (RDW-CV, %) | 13.75±1.52 | 13.09±1.05 | 0.001^*^ |
| Red Cell Distribution Width-Standard Deviation (RDW-SD, fL) | 39.28±3.20 | 37.96±2.74 | 0.009^*^ |
| Platelet (×10^9^ /L) | 363.45±157.41 | 345.27±105.92 | 0.374 |
| CRP (mg/L) | 80.28±52.32 | 78.43±47.39 | 0.828 |
| ESR (mm/h) | 56.9±30.81 | 63.53±28.48 | 0.313 |
| Alanine aminotransferase (ALT, U/L) | 35.17±35.29 | 87.55±162.86 | 0.056 |
| Aspartate aminotransferase (AST, U/L) | 44.84±31.21 | 81.11±157.03 | 0.164 |
| Total bilirubin (umol/L) | 7.52±7.01 | 11.51±15.10 | 0.116 |
| Direct bilirubin (DBIL, umol/L) | 3.76±6.13 | 6.22±12.04 | 0.259 |
| Indirect bilirubin (umol/L) | 3.69±2.84 | 4.87±2.90 | 0.033^*^ |
| Albumin (ALB, g/L) | 37.01±6.74 | 38.96±4.78 | 0.035^*^ |
| Globulin (GLB, g/L) | 30.93±9.18 | 26.73±21.87 | 0.252 |
| Prealbumin (PA, g/L) | 62.00±28.35 | 55.24±28.24 | 0.390 |
| γ-Glutamyl transpeptidase (γGT, U/I) | 53.70±77.99 | 68.87±92.94 | 0.353 |
| Lactate dehydrogenase (LDH, U/L) | 434.11±220.69 | 307.94±142.43 | 0.000^*^ |
| Urea nitrogen (UN, mmol/L) | 3.27±1.38 | 3.34±1.34 | 0.791 |
| Creatinine (Cr, umol/L) | 30.70±8.73 | 26.81±7.63 | 0.006^*^ |
| Serum cystatin C (CysC, umol/L) | 0.93±0.26 | 0.80±0.18 | 0.012^*^ |
| K+ (mmol/L) | 3.99±0.69 | 4.08±0.53 | 0.412 |
| Na+ (mmol/L) | 137.23±4.02 | 135.28±3.01 | 0.001^*^ |
| Cl- (mmol/L) | 102.25±4.59 | 102.12±3.30 | 0.833 |
| Ca2+(mmol/L) | 2.25±0.22 | 2.22±0.14 | 0.299 |
| Mg2+ (mmol/L) | 0.88±0.11 | 0.83±0.08 | 0.003 |
| Total cholesterol (TC, mmol/L) | 3.38±0.72 | 3.25±0.65 | 0.443 |
| Triglyceride (TG, mmol/L) | 1.33±0.46 | 1.36±0.49 | 0.835 |
| HDL-C (mmol/L) | 0.78±0.49 | 0.72±0.30 | 0.497 |
| LDL-C (mmol/L) | 2.20±0.67 | 2.34±0.71 | 0.470 |
| Prothrombin time (s) | 12.97±1.19 | 12.58±1.06 | 0.166 |
| Activated partial thromboplastin time (s) | 36.67±8.80 | 29.63±3.20 | 0.000 |
| Fibrinogen (Fg, mg/d) | 462.83±175.95 | 613.71±119.60 | 0.000^*^ |
| D-dimer (mg/L) | 6.45±8.36 | 1.21±1.10 | 0.009^*^ |
| FDP (mg/L) | 19.74±23.22 | 9.95±14.25 | 0.172^*^ |
| Thrombin time (S) | 16.64±1.68 | 15.61±0.78 | 0.000^*^ |
| Antithrombin III(g/L) | 65.31±30.71 | 83.78±14.43 | 0.034^*^ |
| CTnI (ug/L) | 0.15±0.31 | 0.021±0.028 | 0.025^*^ |
| Mb (ng/mL) | 20.43±11.86 | 7.20±17.67 | 0.018^*^ |
| NT-BNP (pg/ml) | 5152.92±5536.38 | 925.95±2368.25 | 0.019^*^ |

*p < 0.05; CRP, C-reactive protein; KD, Kawasaki diseases; MP, mycoplasma pneumonia; RBC, red blood cell.
